# Evaluation of ruminal methane and ammonia formation and microbiota composition as affected by supplements based on mixtures of tannins and essential oils using Rusitec

**DOI:** 10.1186/s40104-024-01005-8

**Published:** 2024-04-02

**Authors:** Giulia Foggi, Melissa Terranova, Matteo Daghio, Sergej L. Amelchanka, Giuseppe Conte, Simon Ineichen, Monica Agnolucci, Carlo Viti, Alberto Mantino, Arianna Buccioni, Michael Kreuzer, Marcello Mele

**Affiliations:** 1 Department of Agriculture, Food and Environment (DAFE), University of Pisa, 56124 Pisa, Italy; 2 AgroVet-Strickhof, ETH Zurich, Lindau, Switzerland; 3 Department of Agriculture, Food, Environment and Forestry, University of Florence, Florence, 50144 Italy; 4 Centro Di Ricerche Agro-Ambientali “E. Avanzi”, University of Pisa, Pisa, 56122 Italy; 5 School of Agricultural, Forest and Food Sciences HAFL, Bern University of Applied Sciences, Zollikofen, Switzerland; 6 Institute of Agricultural Sciences, ETH Zurich, Lindau, Switzerland

**Keywords:** Additive, Bacteria, Continuous culture, Degradability, Fermentation, Fungi, Greenhouse gas, In vitro, Microbiome, Rumen

## Abstract

**Background:**

Dietary supplements based on tannin extracts or essential oil compounds (EOC) have been repeatedly reported as a promising feeding strategy to reduce the environmental impact of ruminant husbandry. A previous batch culture screening of various supplements identified selected mixtures with an enhanced potential to mitigate ruminal methane and ammonia formation. Among these, Q-2 (named after quebracho extract and EOC blend 2, composed of carvacrol, thymol, and eugenol) and C-10 (chestnut extract and EOC blend 10, consisting of oregano and thyme essential oils and limonene) have been investigated in detail in the present study with the semi-continuous rumen simulation technique (Rusitec) in three independent runs. For this purpose, Q-2 and C-10, dosed according to the previous study, were compared with a non-supplemented diet (negative control, NC) and with one supplemented with the commercial EOC-based Agolin^®^ Ruminant (positive control, PC).

**Results:**

From d 5 to 10 of fermentation incubation liquid was collected and analysed for pH, ammonia, protozoa count, and gas composition. Feed residues were collected for the determination of ruminal degradability. On d 10, samples of incubation liquid were also characterised for bacterial, archaeal and fungal communities by high-throughput sequencing of 16S rRNA and 26S ribosomal large subunit gene amplicons. Regardless of the duration of the fermentation period, Q-2 and C-10 were similarly efficient as PC in mitigating either ammonia (−37% by Q-2, −34% by PC) or methane formation (−12% by C-10, −12% by PC). The PC was also responsible for lower feed degradability and bacterial and fungal richness, whereas Q-2 and C-10 effects, particularly on microbiome diversities, were limited compared to NC.

**Conclusions:**

All additives showed the potential to mitigate methane or ammonia formation, or both, in vitro over a period of 10 d. However, several differences occurred between PC and Q-2/C-10, indicating different mechanisms of action. The pronounced defaunation caused by PC and its suggested consequences apparently determined at least part of the mitigant effects. Although the depressive effect on NDF degradability caused by Q-2 and C-10 might partially explain their mitigation properties, their mechanisms of action remain mostly to be elucidated.

**Supplementary Information:**

The online version contains supplementary material available at 10.1186/s40104-024-01005-8.

## Background

Essential oils (EO) and tannins have drawn the attention of animal nutritionists for decades, as they may be an effective strategy to modulate rumen microbial fermentations based on natural compounds and as such likely to be accepted by the public [1]. The current challenge of climate change has intensified the research toward dietary supplements able to lower ruminal methane and ammonia formation, with the latter resulting in lower urinary N excretion and thus limiting emissions like nitrous oxide from the manure [2]. The EO were initially studied for their suitability to replace antibiotics in animal production [3]. In the meantime, the focus has been put more on environmentally relevant issues, especially lowering methane emission [4–6]. Concerning the dietary tannins, their initially adversely considered properties to inhibit cellulolytic and proteolytic activities in the rumen turned out to be a way to mitigate methane emission and urinary nitrogen excretion [7, 8]. These properties have been confirmed repeatedly [1, 6]. Moreover, tannins, depending on whether they have a hydrolysable or condensed structure, were also described to have a modulatory or inhibitory effect on ruminal biohydrogenation [1]. The findings so far resulted also in some commercial products based either on EO compounds (EOC) or tannin extracts. For instance, the product Agolin Ruminant, containing mainly coriander oil, geranyl acetate and eugenol as EOC [9], was found to reduce both methane yield [10, 11] and ammonia formation [12]. Similarly, the tanniferous extracts from quebracho and chestnut are also commercialised as feed supplements (pure extract or mixture).

Plant active compounds may act in the rumen metabolic pathways at different levels; therefore, the combination of different compounds can serve a useful strategy to modulate rumen microbial fermentation [13]. However, potentially effective supplements have rarely been studied for the additivity of their effect when provided in mixture. Numerous combinations of tannin sources and EOC are possible. For this reason, in our previous study, we performed an extensive short term in vitro screening experiment with the Hohenheim Gas Test, and the results indicated that there was a wide range of responses of different combinations with varying mitigating potential concerning ruminal methane and ammonia formation when comparing them with either tannin extracts or EOC alone [14]. At that time, various sources of EOC and tannin extracts had been selected based on available literature (EOC [15–17]; tannin extracts [1, 18–20]) and compounds not inscribed in the European register of feed additives (Reg. EC 1831/2003) were excluded. However, an in-depth evaluation of the reasons behind the greater mitigation potentials measured with certain mixtures could not be provided with the parameters considered. Additionally, the results described only a preliminary and short-term response [14]. For these reasons, a more sophisticated approach for testing the promising combinations is the semi-continuously operating rumen simulation technique (Rusitec) [21]. This simulator allows the evaluation of nutrient degradability and single gas production in a mid-term fermentation period [22], while meets the need for applying the 3R principles: replace, reduce, refine for experiment purposes [23]. Even though there are limitations in the use of Rusitec in simulating real in vivo conditions [22, 24], it provides useful data in the field of ruminant nutrition and represents a way to better set up in vivo experiments [25].

The aim of the present study was to conduct a more detailed and medium-term evaluation of two mixtures based on tannin extracts and EOC blends with Rusitec, with a particular focus on the effects on ruminal methane and ammonia formation, thus starting to fill the gap in the research on complementarity of active ingredients. The two mixtures selected had turned out to be particularly promising in this respect in the previous screening study [14]. For comparative reasons, a non-supplemented basal diet (NC) and a positive control diet (PC) using a commercial EOC-based supplement with known in vivo effects were included in the experiment. Since most of the mechanisms on rumen microbial and metabolic pathways remained unknown from the previous in vitro screening [14], the effects of the experimental treatments on bacterial, archaeal, and fungal community biodiversity and their relative abundance were also evaluated, to give a better understanding of modifications caused by the supplementation of the tannin-EOC mixtures.

## Methods

### Experimental diets

The four dietary treatments included mixtures Q-2 and C-10 from the screening study [14], NC and PC (Agolin^®^ Ruminant, Agolin S.A., Bière, Switzerland), where EOC dosage was the same with C-10, Q-2 and PC with 7.5 g/kg diet dry matter (DM), equivalent to about 100 mg/L of incubation liquid. Both Q-2 and C-10 contained blends of three essential oils in ratios of 1:1:1 each. These were carvacrol, thymol and eugenol in the case of Q-2 and oregano essential oil, thyme essential oil and citrus peel extract in the case of C-10. The extracts of oregano (*Origanum vulgaris*; main active EOC: carvacrol and b-caryophyllene) and thyme (*Thymus vulgaris*; main active EOC: thymol, p-cimene, c-terpinene, linalool, and carvacrol) were purchased from Italfeed S.r.l (Milan, Italy), while the other EOC sources were obtained from Moellhausen S.P.A. (Milan, Italy). The main EOC declared to be used in Agolin Ruminant are eugenol, geranyl acetate and coriander oil [9]. The mixture treatments in addition contained 10 g/kg of tannin extract, equivalent to 133 mg/L of ruminal medium. These were quebracho, rich in condensed tannins (CT, ≥ 69% of tannins on a DM basis) in Q-2 and chestnut, rich in hydrolysable tannins (HT, ≥ 75% of tannins on a DM basis) in C-10. Tannin extracts were both purchased from Silvafeed ENC powder (Silvateam, Italy). Dosages of tannin extracts and EOC were equivalent to the dosages tested in the previous batch study [14] when expressed per unit of volume of medium (100 mg/L for EOC and 133 mg/L for tannins), as the feed to rumen medium buffer (30 vs. 900 mL) was different in the two approaches.

All supplements were added to 12 g/d of a basal diet formulated to theoretically cover the requirements of a lactating dairy cow of 700 kg body weight and producing 28 kg/d of milk according to the Cornell Net Carbohydrate and Protein System (Nutritional Dynamic System software, RUM&N SAS, Reggio Emilia, Italy). The basal diet was composed by alfalfa hay (415 g/kg DM), grass hay (220 g/kg DM), soybean meal (100 g/kg DM), linseed oil (20 g/kg DM) and concentrate (245 g/kg DM). The latter contained corn flakes (550 g/kg DM), barley (250 g/kg DM), soybean meal (20 g/kg DM), and wheat bran (18 g/kg DM). Since Agolin Ruminant includes other ingredients (hydrogenated sunflower fat, wheat flour, as declared by the commercial datasheet of the producers) besides the EOC-blend, the basal diet was adjusted for treatment PC to result in a similar nutritional composition of the total diet, namely, per kg of DM 132 g crude protein (CP), 89 g ether extract, 496 g neutral detergent fibre (NDF), 340 g acid detergent fibre (ADF), 42 g acid detergent lignin (ADL), and 76 g total ash. Roughages were ground to pass a 5-mm sieve, whereas the other ingredients of the diets were ground to pass a 1-mm sieve.

### Rusitec experiment

A total of three runs were conducted with a Rusitec as described in detail by Soliva and Hess [26] with respect to construction and operation. The Rusitec had eight 1-L fermentation vessels, that was possible to test each of the four different treatments in duplicate per run, adding up to a total of 6 replicates per treatment. The allocation of treatments to the fermenter vessels followed a rotational approach. Each run lasted for 10 d: 5 d were reserved for adaptation and the last 5 d for collecting data and samples.

Rumen fluid was collected from one animal per run, for a total of three lactating rumen-fistulated Original Brown Swiss cows as rumen donors. Donors were fed with a diet composed (g/kg DM) of maize silage (550), grass silage (380), ryegrass hay (20), dairy concentrate (50; UFA-243, UFA AG, Switzerland). Once collected, the rumen fluid was stored anaerobically in sealed pre-warmed bottles and then immediately strained through four layers of medical gauze with a pore size of about 1 mm. McDougall’s solution was prepared as a buffer medium [27], and 100 mL of the buffer was added to each fermenter, previously to 900 mL pure rumen fluid inoculum. Fermentation was conducted as described in detail by Soliva and Hess [26]. Briefly, 900 mL of pure rumen fluid was added to each fermenter. In each inner glass vessel of the fermenters, 2 nylon bags (100 μm pore size) were inserted. One bag contained approximately 70 g of solid rumen content, which helped to inoculate also with ruminal microorganisms attached to the feed, while the other bag contained the experimental diets. The first bag was replaced with a feed-containing nylon bag after 24 h, and the second bag was replaced with another feed-containing bag after 48 h. Each feedbag contained 12 g of basal diet on a DM basis and, depending on the treatment, an additive, as detailed in the Experimental diets section. Subsequently, each feedbag was replaced after 48 h of fermentation, with a total of 9 feedbags fermented for 48 h and 1 feedbag fermented for 24 h (the one inserted the second-to-last day of the run) from each vessel. This process ensured the daily replacement of a fermented bag with a fresh one, maintaining 2 bags in each fermentation vessel each day. The 2 bags in each vessel consistently had a 24-h gap between immersions. On d 1, once rumen fluid and nylon bags had been inserted, the fermenters were closed tightly and kept under nitrogen gas flow (gas flow controller set to 3 L/min) for 3 min to establish anaerobic conditions. This procedure was repeated daily after feedbag replacement. Afterwards, a gas-tight aluminium bag (TECOBAG 8 L, PETP/AL/PE–12/12/75 quality; Tesserau Container GmbH, Burstadt, Germany) was connected to each fermenter to collect the complete fermentation gas. Every day, before disconnecting the bag from the closed circuit, 1.5 L of gaseous nitrogen (30 s at the flux of 3 L/min) was injected into each fermentation vessel to ensure the accumulation of all fermentation gases into the aluminium bag. The buffer was continuously infused into the fermenters at a rate which resulted in an average incubation liquid outflow of 495 mL/d/fermenter. This was equivalent to a dilution rate of about 50%/d.

### Sampling and laboratory analyses

Throughout the whole experimental period and always 3 h before exchanging the feed bags, a fixed amount of 10 mL of incubation liquid was collected daily directly from the fermenters. On those rumen liquor samples, the pH (pH Meter 913, Metrohm Suisse SA, Zofingen, Switzerland) was measured. On the same samples and during the last 5 d, in addition ammonia concentration (NH_3_-N) was measured with an ammonia-selective gas membrane electrode (6.0506.010 connected to pH Meter 632, Metrohm SA, Zofingen, Switzerland), and protozoa and bacteria counts were determined with Bürker counting chambers with a depth of 0.1 mm or 0.02 mm, respectively (BlauBrand, Wertheim, Germany). To determine the production and composition of volatile fatty acids (VFA) in the incubation liquid, 4 mL were centrifuged at 2,600 × *g* for 5 min at 4 °C, and 2 mL of the supernatant was frozen (–20 °C) for later analysis with HPLC following Shahab et al. [28]. On the last day of fermentation, rumen fluid was also sampled and immediately frozen at –80 °C for later microbiota composition analysis.

For the whole duration of the experimental period (5 d), the total amount of fermentation gas produced during 24 h was measured by the water displacement technique [26]. To obtain the total gas produced (GP) with fermentation, the amount of nitrogen gas injected in 30 s at the flux of 3 L/min (1.5 L) was subtracted from the measured gas amount in the aluminium bag. A total of 5 aluminium bags containing gas were collected for each run and fermentation vessel. The gas was sampled with a gas-tight Hamilton syringe and analysed for concentrations of CH_4_, CO_2_ and H_2_ with a gas chromatograph (GC-TCD 6890N, Agilent Technologies, Wilmington, NC, USA). From the concentrations measured, the volume of gases of interest was obtained (CO_2_, CH_4_ and H_2_), and their concentrations were then calculated in relation to the sole fermentation GP volume instead of the GP volume together with the volume of the gaseous nitrogen infused.

The bags with the feed residues fermented for 48 h during the experimental period were washed in a washing machine with cold water without spinning and then frozen at –20 °C prior to freeze-drying. For each run and fermentation vessel, the feedbags analysed for compositional analysis were the four fermented from d 5 to d 10. The feedbag inserted on d 9 was excluded as it was fermented only 24 h. Before chemical analysis, the feed (pre-dried at 60 °C for 48 h) and freeze-dried residues were ground with an ultra-centrifugal mill (Model ZM 200, Retsch GmbH, Hann, Germany) to pass through a 1-mm sieve. The DM content was then determined by oven drying at 105 °C until weight was stable. Total ash content was subsequently measured after ashing at 550 °C for 3 h. Organic matter (OM) was calculated as the difference between DM and total ash. The nitrogen content was determined with the Kjeldahl method, and CP was calculated as N × 6.25. An Ankom XT10 Extractor (Astori Tecnica, Brescia, Italy) was used to determine ether extract with the AOCS Official Method Am 5–04 method [29]. The contents of NDF, ADF and ADL were analysed with the filter bag technique, according to the Van Soest et al. [30] protocol and using the Ankom Fibre Analyzer A200 (Astori Tecnica Brescia, Italy). The NDF analysis included a preceding incubation with α-amylase. All samples were analysed in duplicate.

### DNA extraction, sequencing, and bioinformatics

DNA was extracted from 500 μL of incubation fluid by using the Fast DNA Spin for soil kit (MP Biomedicals, Solon, OH, USA) following the manufacturer’s protocol modified as previously reported [31]. The V3–V4 region of the 16S rRNA gene was amplified with Pro341F and Pro805R primers [32] for taxonomic characterization of bacterial and archaeal communities. The 26S ribosomal large subunit (LSU) was amplified with LS2-MF and NL4 primers [33, 34] for the characterization of the fungal communities. Amplicons preparation and sequencing were performed at IGA Technology Services (Udine, Italy) by MiSeq Illumina (Illumina, Inc., San Diego, CA, USA) using a 300 bp 2 paired-end protocol.

Bioinformatic elaborations were performed as follows: primers were removed using cutadapt v3.5 [35]. Further bioinformatics elaboration was performed using usearch v11 [36]. Forward and reverse reads were merged, and a quality filter was applied (maximum expected error threshold = 1.0). The reads were dereplicated and error-correction of amplicon reads was performed using UNOISE algorithm [37] with default parameters to generate the zero-radius operational taxonomic units (zOTUs) and chimera were removed. The reads were mapped against the zOTUs with default parameters. Taxonomic assignment for each zOTU was performed against the RDP database v18 (16S rRNA gene) [38] and against the RDP LSU (LSU) [39] in R 4.2.1 (R Core Team) using the assignTaxonomy function of dada2 package v1.24.0 [40] with 80% confidence. For the 16S rRNA gene, a total of 1,111,766 high-quality sequences were obtained with an average of 46,324 ± 5,443 sequences per sample (average ± standard error). For the LSU, a total of 593,570 high-quality fungal sequences were obtained with an average of 24,732 ± 1,965 sequences per sample (average ± standard error).

### Calculations and statistical analysis

Degradability of DM (dDM), OM (dOM), NDF (dNDF) and CP (dCP) was calculated from the difference between supply and residue after a 48 h of incubation time. In addition, the degradability of OM and NDF was related to DM supply (dOM_DM_ and dNDF_DM_). Production of CH_4_ and CO_2_ was calculated as the product of GP and their corresponding concentration in the gas phase. CH_4_ yield was calculated per gram of DM supplied and per gram of OM and NDF disappeared during 48 h of fermentation, and, finally, per mole of VFA produced.

All data were analysed with a linear mixed model considering the fixed effects of treatment (NC, PC, C-10, and Q-2), period (d 6, 7, 8, 9 and 10), and the interaction treatment × period as fixed effects, the run as random effect, and the residual error (JMP 16.0, SAS Institute Inc., Cary, NC, USA). Differences among treatment means of the ruminal fermentation data were declared significant at *P* < 0.05 based on a HSD-Tukey as post hoc analysis test. Effects of period and its interaction with treatment are not displayed in tables; however, when such effects were significant, they were reported in the text. No interaction effect treatment × period resulted significant.

The 16S rRNA gene and the LSU sequencing data were further processed using the vegan package, version 2.6.2 [41] in R 4.3.0 (R Core Team). The Chao1 index, the ACE index, the Shannon diversity index, and the Simpson index were calculated to estimate the alpha-diversity, a Kruskal–Wallis test was performed to detect significant differences between the conditions and multiple comparison was performed by a Dunn test (*P*-values were corrected using the Benjamini–Hochberg adjustment). The alpha-diversity was estimated on a randomly rarefied dataset (21,836 sequences per sample for the 16S rRNA gene and 9,134 sequences per sample for the LSU). A non-metric multidimensional scaling (NMDS) and a permutational multivariate analysis of variance (PERMANOVA) based on Hellinger transformed zOTUs abundance data were performed using the metaMDS and the adonis2 functions, respectively. Both the NMDS and the PERMANOVA were performed on the weighted UniFrac distances. The taxa with a different relative abundance between the conditions were identified by a Kruskal–Wallis test and multiple comparison was performed by a Dunn test where *P*-values were corrected using the Benjamini–Hochberg adjustment.

## Results

### Fermentation profiles and nutrient degradability

The pH of the incubation fluid ranged between 7.02 and 7.11, with significantly lower values obtained with NC and C-10 than with Q-2 and PC (Table 1). Also, the incubation period influenced the pH, which significantly declined after d 7 of fermentation (from 7.09 to 6.98 on d 10, *P* < 0.01, data not shown in tables). Compared to NC, the NH_3_ concentration was lowered by the supplements, particularly by treatments Q-2 and PC (by 37% and 34%, respectively), followed by treatment C-10 (−25.5%), but it was not affected by the period. Both Q-2 and PC also decreased the VFA formation by 14% and 17%, respectively. This decrease was mainly caused by the lower amounts of acetate (−16%) with both Q-2 and PC and propionate (−25%) with Q-2 and −37% with PC (data not shown in tables). Accordingly, the acetate-to-propionate ratio varied from 2.22 with NC to 2.96 with PC (Table 1), with intermediate ratios found with C-10 (2.36) and Q-2 (2.49). Regarding the most abundant VFA, the molar VFA profile notably differed between NC on the one hand and Q-2 and PC on the other hand. In comparison to NC, the lower molar proportion of acetate (only Q-2) and propionate (both PC and Q-2) were compensated by higher molar proportions of butyrate. The differences between NC and C-10 were smaller; however, C-10 showed a slightly lower propionate proportion (−6%) and a slightly higher butyrate proportion (+8%) than NC. Concerning less abundant VFAs, NC and C-10 reported minor differences, except for valerate proportion, which was significantly lower in C-10 and Q-2 compared to NC and PC. Iso-butyrate and iso-valerate proportions showed slightly higher concentrations in PC and Q-2 treatments compared to C-10 and NC; however, when the absolute concentrations (mmol/L) of branched-chain VFA (BCFA) were considered, PC and Q-2 did not have higher (PC) or even a significantly lower concentration (Q-2) of BCFA (mmol/L) compared to both NC and C-10 (Table 1). The effects of fermentation time on VFA were limited to the acetate molar proportion, which decreased from d 6 to 10, while the valerate and the iso-valerate molar proportion increased (all *P* < 0.001, data not shown in tables).

**Table 1:** Effect of the tannin-essential oil mixtures Q-2 and C-10 compared to negative control (NC) and positive control (PC) on ruminal fermentation variables

Item	**NC**	**PC**	**Q-2**	**C-10**	**SEM**	***P*-value**
pH	7.02^b^	7.11^a^	7.08^a^	7.00^b^	0.028	< 0.001
NH_3_-N, mmol/L	10.52^a^	6.94^c^	6.67^c^	7.84^b^	0.927	< 0.001
Volatile fatty acids, mmol/L	82.4^a^	68.2^b^	70.7^b^	81.4^a^	1.35	< 0.001
Branched-chain volatile fatty acids, mmol/L	5.23^a^	5.21^ab^	4.91^b^	5.29^a^	0.255	0.006
Proportions of volatile fatty acids, % of total						
Acetate	49.16^a^	49.52^a^	48.09^b^	49.39^a^	1.030	< 0.001
Propionate	22.69^a^	17.24^d^	19.64^c^	21.42^b^	2.303	< 0.001
iso-butyrate	1.33^b^	1.60^a^	1.54^a^	1.32^b^	0.178	< 0.001
Butyrate	15.61^c^	19.30^a^	19.44^a^	16.86^b^	2.600	< 0.001
iso-valerate	5.06^c^	6.07^a^	5.40^b^	5.19^bc^	0.200	< 0.001
Valerate	6.15^ab^	6.26^a^	5.88^bc^	5.82^c^	1.033	< 0.001
Acetate:propionate ratio	2.22^c^	2.96^a^	2.49^b^	2.36^b^	0.220	< 0.001
Ruminal degradabilites						
Dry matter (dDM), %	59.5^a^	55.2^b^	57.8^a^	58.7^a^	0.84	< 0.001
Organic matter (dOM), % of supply	59.2^a^	51.6^c^	55.4^b^	56.7^b^	0.91	< 0.001
Organic matter (dOM_DM_), % of dry matter (DM) supply	45.7^a^	42.6^b^	43.8^b^	44.4^ab^	0.72	< 0.001
Neutral detergent fibre (dNDF), % of supply	36.2^a^	31.2^b^	25.5^c^	28.2^bc^	1.67	< 0.001
Neutral detergent fibre (dNDF_DM_), % of DM supply	17.6^a^	15.5^b^	11.6^c^	12.9^c^	0.80	< 0.001
Crude protein (dCP), % of supply	84.9^a^	82.5^b^	82.9^b^	83.6^b^	0.42	< 0.001
Protozoa, 10^3^/mL	131^a^	4^b^	118^a^	155^a^	25.9	< 0.001
Bacteria, 10^9^/mL	1.11^b^	1.40^ab^	1.51^a^	1.23^ab^	0.160	< 0.01

^a–d^Means not carrying a common superscript are significantly different at *P* < 0.05

Compared with NC, only supplement PC decreased the ruminal degradability of DM (−7.2%), whereas the other supplemented treatments decreased that of OM (Table 1). The highest reduction of dOM (% of OM supply) was caused by PC (−12.8%), followed by Q-2 (−6.4%) and C-10 (−4.2%). All supplements decreased the CP degradability to an average extent of about 2.2% compared to NC. Treatments Q-2 and C-10 markedly reduced the degradability of NDF, either when related to DM (−34% and −27%, respectively) or to NDF supplied (−30% and −22%), whereas the decline caused by PC was less pronounced (−12% and −14% when related to DM and NDF, respectively). The PC was the only treatment that drastically reduced the number of protozoa (−97% in comparison to NC, Table 1), regardless of the slight variation during the incubation period (*P* = 0.04, data not shown in tables). Bacteria counts were enhanced by supplementation of Q-2 compared to NC, but they were not affected by the period.

### Production of methane and other gases

The total daily GP slightly decreased with C-10 and PC in comparison to NC (−6% on average, Table 2). A similar pattern was observed also for the daily CH_4_ production, which was significantly reduced by C-10 and PC by 12% and 13%, respectively. Supplement Q-2 only numerically reduced the daily CH_4_ production (−7%) compared with NC. The proportion of CH_4_ in the total gas did not vary across treatments. Meanwhile, the CO_2_ proportion of total gas was lowest in treatment PC, followed by Q-2, then C-10, with the highest proportion observed in the NC treatment (Table 2). Consequently, the CH_4_:CO_2_ ratio was higher with Q-2 and PC supplements. Methane yield per gram of diet DM was reduced significantly by C-10 (−12% in comparison to NC) but not by Q-2. Any additive caused a significant reduction of CH_4_ yield per gram of disappeared OM or per mole of produced VFA. CH_4_ yield per gram of disappeared NDF was higher with Q-2 and tended to be higher with C-10 in comparison to NC and PC. Production of H_2_ and its proportion of the total gas was the smallest for PC treatment (Table 2).

**Table 2:** Effect of the tannin-essential oil mixtures Q-2 and C-10 compared to negative control (NC) and positive control (PC) on production, composition and yield of ruminal gases

Item	**NC**	**PC**	**Q-2**	**C-10**	**SEM**	***P*-value**
Daily production						
GP, L	3.41^a^	3.19^b^	3.26^ab^	3.20^b^	0.052	0.025
CO_2_, L	1.40^a^	1.18^b^	1.24^ab^	1.24^ab^	0.044	0.005
CH_4_, mL	187^a^	164^b^	175^ab^	166^b^	5.4	0.018
H_2_, mL	6.58^a^	1.54^b^	5.72^a^	4.69^a^	2.189	< 0.001
CH_4_/CO_2_, mL/L	135^c^	139^b^	144^ab^	134^c^	1.4	< 0.001
% of GP						
CO_2_	40.9^a^	36.5^c^	37.4^bc^	39.6^ab^	0.77	< 0.001
CH_4_	5.52	5.09	5.40	5.32	0.112	0.119
H_2_	0.19^a^	0.05^b^	0.17^a^	0.14^a^	0.063	< 0.001
CH_4_ yield						
mL/g dry matter	14.1^a^	12.5^ab^	13.1^ab^	12.4^b^	0.41	0.029
mL/g disappeared organic matter	31.0	29.4	30.1	28.1	1.02	0.287
mL/g disappeared neutral detergent fibre	82.5^b^	83.0^b^	118.4^a^	100.5^ab^	5.45	< 0.001
mL/mmol volatile fatty acids	2.21	2.23	2.41	2.06	0.101	0.173

^a–c^Means not carrying a common superscript are significantly different at *P* < 0.05. *GP* Gas produced from ruminal fermentation, with the gaseous N_2_ infused into the gas bag subtracted

### Taxonomic composition of the bacterial and fungal communities

**Fig. 1:**
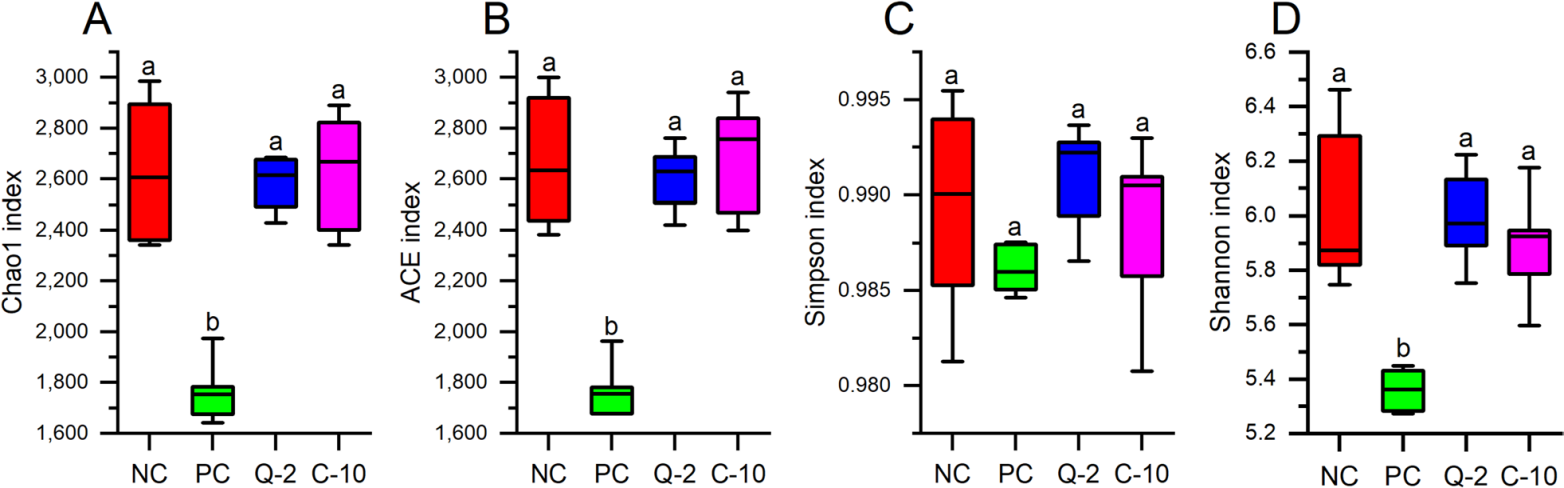
Boxplots of the prokaryotes diversity indices of the rumen microbiota of treatments. **A** Chao1 index. **B** ACE index. **C** Simpson index. **D** Shannon index. NC, negative control; PC, positive control; Q-2, supplement Q-2; C-10, supplement C-10. ^a,b^Boxplots of treatments without common letter are significantly different at *P* < 0.05

**Fig. 2:**
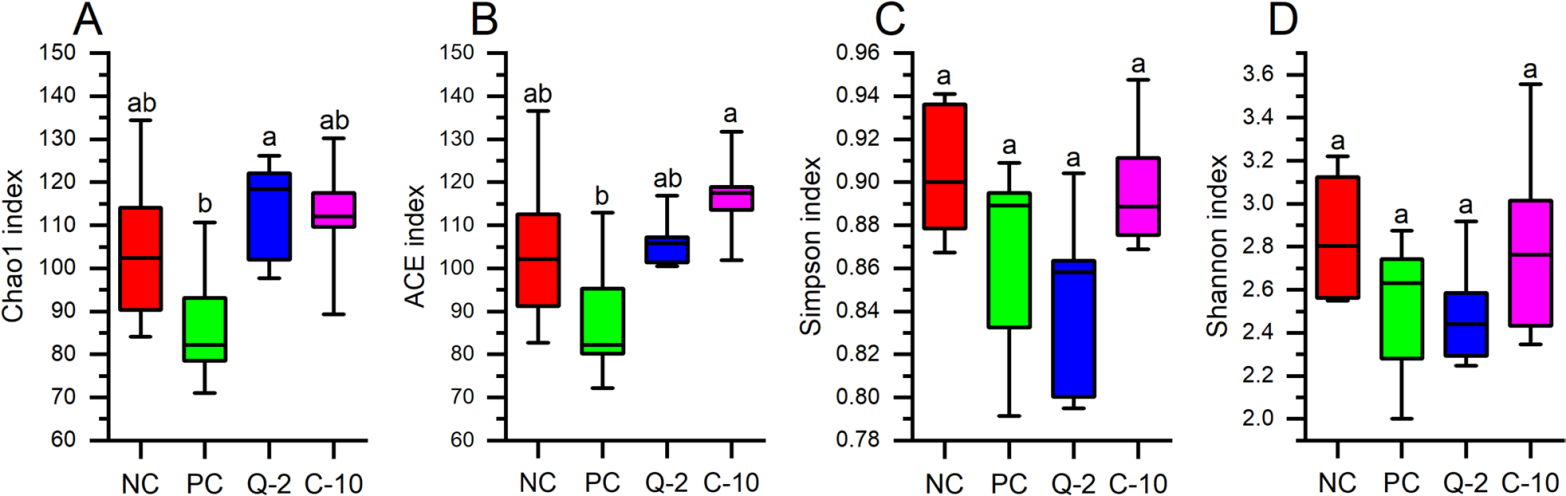
Boxplots of the fungal diversity indices of the rumen microbiota of treatments. **A** Chao1 index. **B** ACE index. **C** Simpson index. **D** Shannon index. NC, negative control; PC, positive control; Q-2, supplement Q-2; C-10, supplement C-10. ^a,b^Boxplots of treatments without common letter are significantly different at *P* < 0.05

**Fig. 3:**
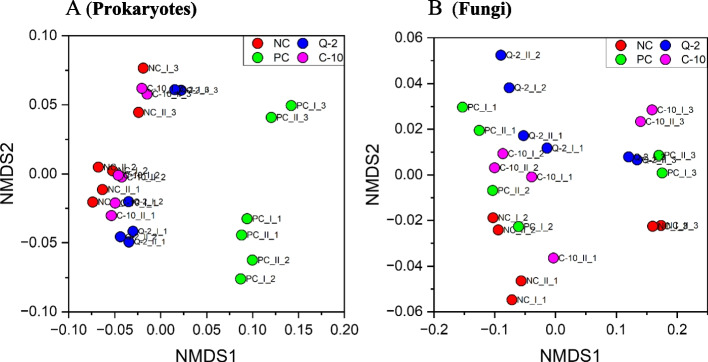
Non-metric multidimensional scaling (NMDS) plot based on weighted UniFrac distances of bacterial (**A**) and fungal (**B**) communities. NC, negative control; PC, positive control. Roman number indicate different samples within a run and Arabic number indicate the run

For the microbial communities in the rumen fluid of the four treatments, as characterized by high-throughput sequencing of 16S rRNA and LSU gene amplicons, respectively, all rarefaction curves reached the plateau (Additional file 1: Fig. S1 and S2), confirming that the sampling depth was sufficient to describe the biodiversity within the dataset. The treatment PC clearly induced a reduction of the prokaryote’s richness in comparison to other treatments, as it was indicated by the Chao1 and ACE indices (Fig. 1). On the contrary, bacterial evenness was less affected by PC, as indicated by Simpson index, but a lower Shannon diversity index was calculated [42]. The fungal richness of PC samples differed from that of the other supplemented treatments, namely to Q-2, considering the Chao1 index and to C-10, the ACE index, but it was not different from NC (Fig. 2). Fungal evenness was not affected by the treatment (Fig. 2). The NMDS plot put in evidence differences in the composition of prokaryotes communities between PC and the three other treatments (among which Q-2 seemed slightly different as well), on NMDS 1, and between run 3 and the other runs, on the NMDS 2 (Fig. 3). The PERMANOVA calculated on the weighted UniFrac distance matrix confirmed such a pattern (*R*^2^ = 0.372, treatment effed had a *P* < 0.001). The NMDS plot for fungal communities showed similar differences between run 3 and the other runs, on NMDS 1, and tended to separate between Q-2 and PC treatment, on the NMDS 2 (Fig. 3).

#### Prokaryote abundances

**Fig. 4:**
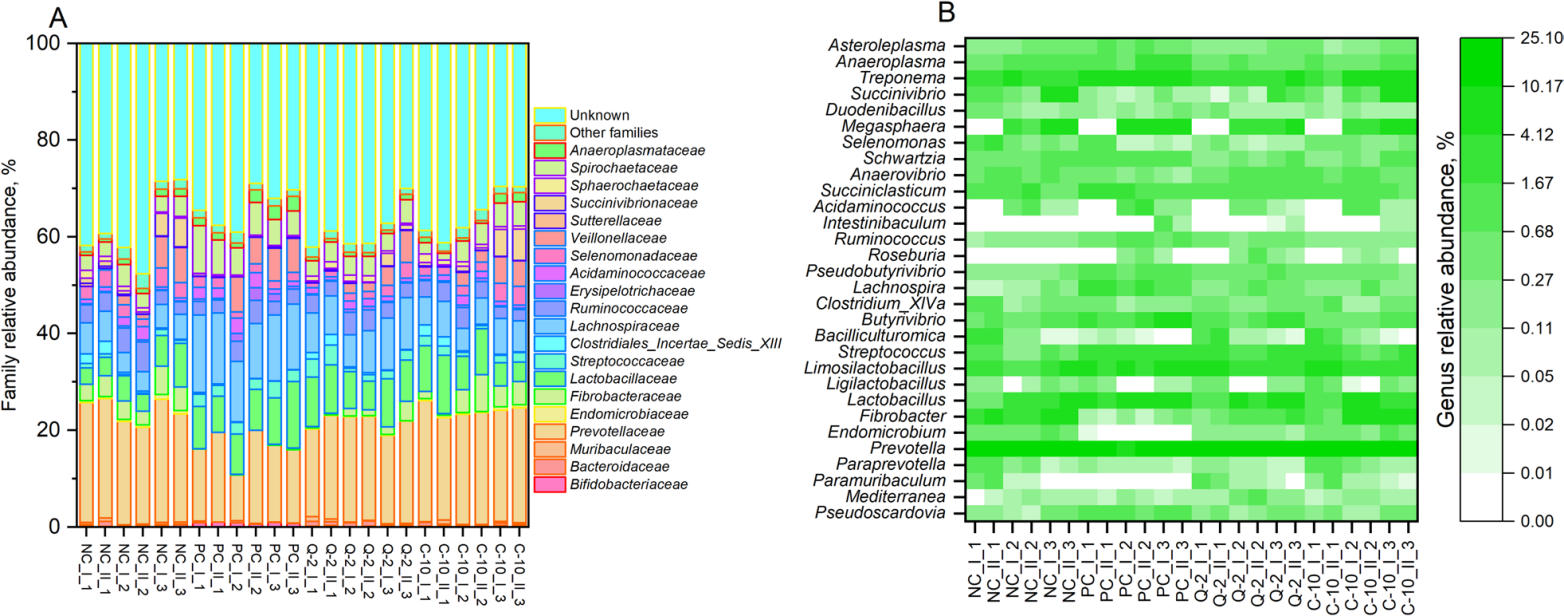
Composition of prokaryotes communities at the family level (**A**) and genus level (**B**). NC, negative control; PC, positive control. Roman number indicate different samples within a run and Arabic number indicate the run

Data concerning family and genus abundances are reported in Supplementary material (Table S1–4). In total, 20 families were classified. The family Prevotellaceae (phylum Bacteroidetes) was the most abundant (20% relative abundance on average, with a range between 15% and 23%), followed by the families Lachnospiraceae and Lactobacillaceae, with a relative abundance average of 8.4 ± 3.8 and 7.7 ± 1.8, respectively (Table S1 and Fig. 4A).

The other classified families made up 25.2% in total, the unclassified families 36.3%. Overall, 30 genera were classified. The most abundant genera were *Prevotella* (phylum Bacteroidetes, average 19.4%, range 14.8%‒21.9%), followed by *Treponema* (phylum Spirochaetes, average 4.4% ± 1.5%) (Fig. 4B). Four other genus groups, namely *Limosilactobacillus* (phylum Firmicutes), *Lactobacillus* (phylum Firmicutes) and *Megasphaera* (phylum Firmicutes) had an average abundance of 3%–4% (Table S2).

The relative abundances of 11 families were significantly affected by the treatment (Table S1). Overall, C-10 did not differ in terms of family abundances from Q-2 and NC. Instead, Q-2 had lower relative abundances of Endomicrobiaceae and higher abundances of Lachnospiraceae, and Streptococcaceae compared to NC. Treatments NC and PC differed in relative abundances of 9 families. Endomicrobiaceae, Fibrobacteraceae, Muribaculaceae, Prevotellaceae, were less abundant in PC than NC, whereas Bifidobacteriaceae, Erysipelotrichaceae, Lachnospiraceae, Streptococcaceae and Spirochaetaceae were more abundant in PC. When compared to other supplemented treatment, PC basically reported the same differences as with NC in comparison to C-10, whereas fewer differences regarded PC and Q-2. Relative family abundances in PC compared to Q-2 were lower for Sphaerochaetaceae but higher for Bifidobacteriaceae, Streptococcaceae and Spirochaetaceae.

Treatments significantly affected the relative abundance of 14 genera (Table S2). Again, C-10 compared to NC or to Q-2 did not show significant differences in terms of genus abundances. Relative genus abundance of Q-2 and NC differed, instead, for only 4 genera, namely: *Endomicrobium*, higher in NC than Q-2, and *Ruminococcus*, *Streptococcus*, and *Pseudobutyrivibrio* higher in Q-2 than NC. A higher number of dissimilarities was observed for genus abundance of PC, which reported a higher abundance of *Treponema* and *Pseudoscardovia* and a lower abundance of *Paramuribaculum*, in comparison to all other treatments. Moreover, 7 additional differences of genus abundance separated PC from NC and C-10. Among others, *Prevotella* (−32%) and *Fibrobacter* (−97%) were significantly less abundant in PC compared to C-10 and NC.

Relative abundances of Euryarchaeota phylum, which represented the total of the Archaea in the present study, were lowered in treatment PC in comparison to Q-2 but not in contrast to the other treatments (Fig. 6).

#### Fungal abundances

**Fig. 5:**
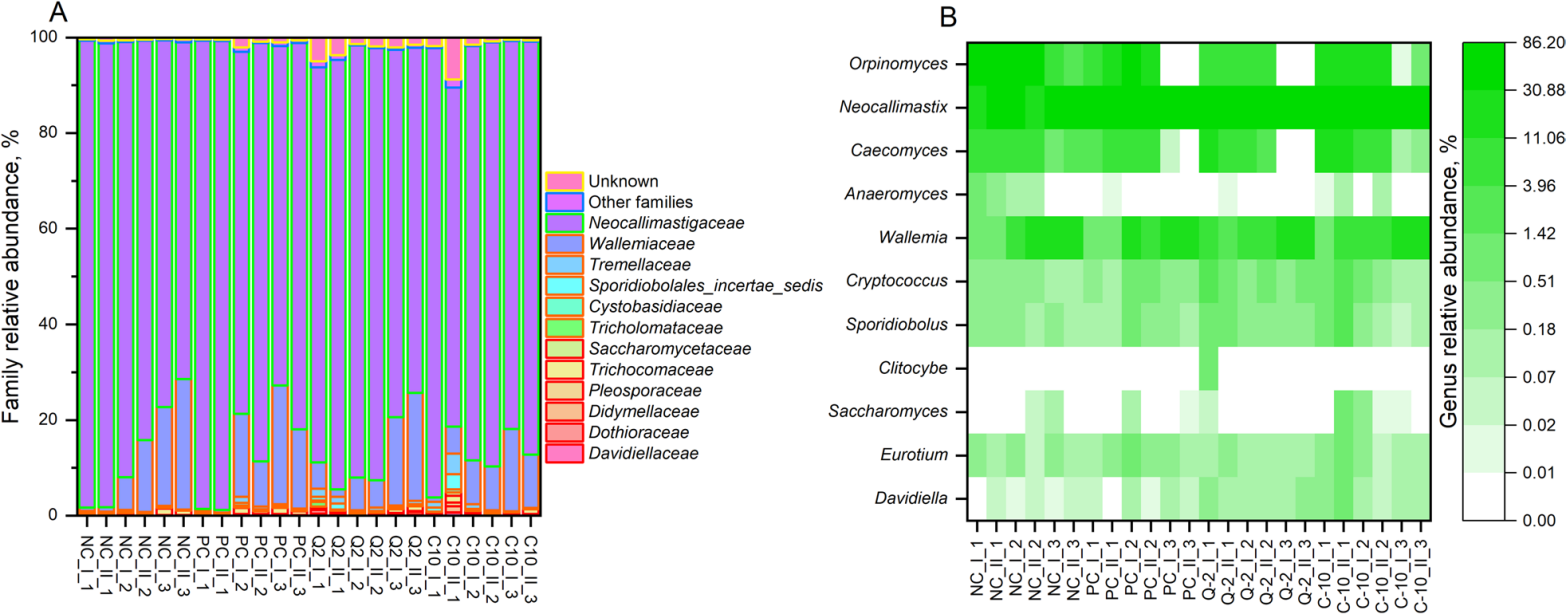
Composition of fungal communities at the family level (**A**) and genus level (**B**). NC, negative control; PC, positive control, Q2, supplement Q-2; C10, supplement C-10. Roman number indicate different samples within a run and Arabic number indicate the run

A total of 12 fungal families were classified (Table S3). The family most present in all samples was Neocallimastigaceae with an average abundance of 84.9% ± 1.0%, followed by Wallemiaceae family, which had an average abundance of 10.6% ± 1.4% (Table S3 and Fig. 5A). Accordingly, the most abundant genera, of the total 11 classified groups, were *Neocallimastix* and *Orpinomyces* (both belonging to family Neocallimastigaceae), with an average abundance of 55.8% ± 12.3% and 14.3% ± 10.6%, respectively, followed by *Wallemia* (Wallemiaceae family) with an average abundance of 10.6% ± 1.4% (Table S4 and Fig. 4B). Significant differences among family abundances were observed only for families with relative abundance less than 1%: Davidiellaceae, Didymellaceae and Tremellaceae highly abundant in Q-2 compared to NC (Table S3). Concerning fungal genus abundances, *Neocallistomastix*, *Cryptococcus* (family Sporidiobolaceae) and *Davidiella* (family Davidiellaceae) were more abundant in Q-2 than NC (Table S3).

## Discussion

Currently, only a few studies focused on the use of a mixture of compounds as supplements instead of testing the single compounds, building on a potential complementary and thus associative effect. Accordingly, blends of EOC [16], a mixture of tannin and saponin extracts [43], mixtures of different types of tannin extracts [44, 45], or mixtures of EOC and tannins [14] were found to be more efficient in reducing methane or ammonia production compared to the supplementation of the individual compounds. Since the dosage and proportion between the active compounds is one of the main issues in the evaluation of the effectiveness of natural compounds as a mitigating strategy [17], the dosages of tannins and EOC blends supplied were adopted from the previous short-term in vitro experiment, expressed as gram per unit of the incubation liquid (133 mg/L of tannin extracts in addition to 100 mg/L of EOC blend; [14]). It has to be noted that the ratio of diet to incubation liquid might differ between in vitro systems, meaning that the dosage per unit of DM can be different. In the present study, the dosage per unit of DM was halved compared to the previous experiment (10 instead of 20 g tannin extracts and 7.5 instead of 15 g EOC blend per kg diet DM). The variability of the feed-to-medium ratio between the different in vitro setups has been previously considered [46], but still, this aspect has been poorly investigated in terms of determining how the supplements are dosed, and further research is needed in this sense.

To evaluate the efficacy of Q-2 and C-10, the promising mixtures selected [14], we included both a negative control and a positive control. The latter consisted of a commercial mixture of EOC that had been previously shown with a meta-analysis [10] to be able to reduce methane emissions under in vivo conditions. Therefore, and for mechanistic reasons, the effects of the newly formulated supplements are discussed separately in the following sections, in comparison to NC (basic efficiency of the mixtures) and PC (comparative effects with a known agent; here blend of EOC).

### Diets Q-2 and C-10 vs. negative control diet

The usage of tannins as supplements has been repeatedly reported to adversely affect the methanogenic and fibrolytic activities in the rumen under in vivo and in vitro conditions [1, 47, 48]. In particular, tannins disrupt more Gram-positive than Gram-negative bacteria [1]. Essential oils were described as having a wide spectrum of anti-microbial activities. Due to their lipophilic nature and generally low molecular weight, they can penetrate the outer membranes of Gram-positive bacteria and protozoa and even the external membranes of Gram-negative bacteria (only low molecular weight EO) and thus cause disruption [13, 49].

To the best of our knowledge, only one other in vitro previous attempt (apart from Foggi et al. [14]) has been made to test the effect of a mixture of tannins (chestnut extract) in combination with an EO blend (bioflavonoids extracted from olives) [50]. In that study, the in vitro fermentation was conducted using the biochemical methane potential assay. The inoculum was not rumen fluid, but anaerobic mud. Moreover, the substrate fermented constituted exclusively of anhydrous glucose and no fibrous material. Therefore, the comparability with ruminal conditions is very limited. In the literature, also the availability of in vivo studies is limited. Recently, Atzori et al. [51] supplemented 1 g/d of a blend of essential oils, bioflavonoids and chestnut tannins to Sarda sheep and reported a promising mitigating effect on methane yield (−13%), even though no effect was observed on absolute methane production.

In the present study, the supplement Q-2, based on quebracho tannins and carvacrol, thymol and eugenol as EOC, was effective in substantially mitigating ammonia formation (−37%). Its reduction potential was larger than the one that had been found with Q-2 in the short-term in vitro study [14]. The effect of supplement C-10, based on chestnut tannins and EO from oregano, thyme and citrus peel, on ammonia production was also significant, but at lesser extent (−26%, similar to the 25% found previously [14]). Ammonia in the rumen can be produced by various microbial species with low specificity. In addition, a small group of microbes producing ammonia have a high specificity for substrates containing nitrogen, the so-called hyper ammonia producers [3]. In literature, there is evidence about the efficacy of EO (a blend [3] or oregano EO [52] to selectively reduce some hyper ammonia producers. Nevertheless, other EO blends seemed to be inefficient in inhibiting hyper ammonia producers (e.g., *Clostridium aminophilum* [5]). There might be several reasons for the greater efficiency of Q-2 compared to C-10 in mitigating ruminal ammonia production in the present study. Accordingly, a synergistic effect of specific EOC blend components of Q-2 and quebracho tannins might have a mitigating effect on specific bacteria populations. Interestingly, a lower relative abundance of *Endomicrobium* was found in the fluid from Q-2 treatment. Since such a bacterium was described as having an unusual nitrogenase, the decreasing of its relative abundance in the microbiota might be at least partially responsible for the lower ammonia production in Q-2, as similarly found by Mavrommatis et al. [53] supplementing a marine microalga. Another reason for lower ammonia concentration in Q-2 and C-10 incubation fluid might be the lowering of dCP compared to NC treatment. However, a different efficiency in reducing CP degradability between Q-2 and C-10 was, however, not found in the present study. Despite that, Q-2 rumen liquor had a lower BCFA concentration (mmol/L), which together with the higher depression of ammonia, suggested a reduced deamination of certain amino acids (leucine and valine) compared to C-10 [54].

**Fig. 6:**
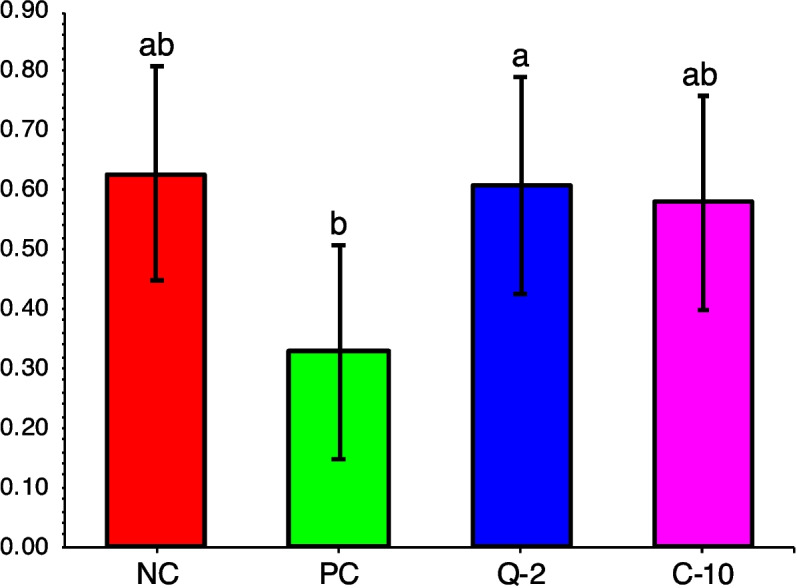
Relative abundances of *Euryarchaeota phylum* (total of the Archaea in the present study). ^a,b^Different superscript letters indicate significant differences (*P* < 0.05) between treatments. NC, negative control; PC, positive control

In the present study, there was only a numerical decline in methane formation by 7% with Q-2, whereas in the previous in vitro experiment the reduction in methane formation accounted for 14% [14]. On the other hand, the significant methane mitigation caused by C-10 by 12% was more similar to the one previously found (14%), even though archaeal abundance did not differ (Fig. 6), in accordance with what was reported previously when CT or HT were supplemented in vitro [55]. Overall, there were no differences in the microbial community (at least at genus level), in support of the different methane reduction extent. As such, the outcomes are not coherent with a previous study, in which a target genus (i.e., *Prevotella*) was found as greater abundant in rumen liquor of low buffalo emitters [56] or in rumen liquor supplemented with CT or HT tannins [55]. However, direct action against methanogens mediated either by EOC [16], by fractions obtained by HT hydrolysis [20] or by their combination cannot be fully excluded, since 16S sequencing may fail in identifying less abundant taxa but biologically meaningful [57].

The fibre-degrading microorganisms are the main responsible for the hydrogen production which, in turn, is converted to methane by methanogenic archaea. Tannins, especially CT [58], are known to decrease fibre degradability. As such, the adverse effect of both mixtures on fibre degradability (Table 2) might have contributed to the methane mitigation in the present study. Indeed, CH_4_ produced per unit of disappeared NDF was higher for both mixtures in comparison to NC. However, Q-2 and C-10 did not result in a lower relative abundance of characteristic fibrolytic bacteria (*Fibrobacter*, *Butyrivibrio, Prevotella*; [49]). It can be only assumed that the reduction in the relative abundance of fungal *Orpinomyces* (in tendency with both Q-2 and C-10, Table S4) might indicate one reason for the reduction of fibre degradability. Indeed, a tendency to adversely affect rumen anaerobic fungi, which primarily degrade fibre components, by supplementing eucalyptus oil and mulethi root aqueous extract as an additive was previously described in rumen liquor of buffalo [59] and in vitro with the supplementation of chestnut or quebracho extracts [20]. However, in contrast to the above, the supplements (significant only for Q-2) even enhanced the abundance of *Neocallimastix*; thus, it is evident that several factors concurred to determine the outcome. Moreover, an effect on unclassified fibrolytic bacteria or anaerobic fungi cannot be excluded.

The more pronounced reduction of fibre degradability with the extract containing CT (Q-2) compared to HT (C-10) was associated with the lowest production of acetate (the main end-product of fibrous degradation) as previously reported [18]. Consequently, with Q-2 less total VFA were produced, partially confirming what was previously found in our in vitro screening study [14]. Similar to the present study, Buccioni et al. [60] reported that quebracho extract decreased acetate production along with the total VFA produced in the rumen of dairy sheep. The lower acetate production did not decrease the hydrogen concentration in the gas phase of Q-2 treatment sufficiently enough to clearly affect methanogenesis [61], as confirmed by the data about methane production for Q-2 treatment.

### Diets Q-2 and C-10 vs. positive control diet

Outcomes from various in vitro studies may vary largely [62]. The inclusion of a common supplement as a positive control might thus help in comparing studies with different in vitro setups. Especially monensin was recommended for this purpose in studies with dairy [55], beef cattle [63], and in vitro continuous culture [64]. However, the active principle of monensin is far from that of phenolic compounds or EO. Therefore, in the present study, Agolin Ruminant, a commercial additive also based on a blend of EOC, namely coriander oil, geranyl acetate and eugenol, was employed as a positive control. In order to facilitate comparability, the dosage of the EOC from this supplement was kept the same as with Q-2 and C-10.

When compared with PC, Q-2 and C-10 had a similar effect on either ammonia (−37% by Q-2, −34% by PC) or methane emissions (−12% by C-10 and PC). This was expected being Q-2 and C-10 formulated with the same criteria (i.e., tannin extract + 3-way EOC) and having some ingredients in common (i.e., carvacrol and thymol were included in both Q-2 and C-10). Unlike PC, Q-2 and C-10 did not affect either protozoa count or hydrogen concentration in the fermentation gas. This observation suggests different mechanisms of action of Q-2 and C-10 vs. PC. In contrast to what was previously measured in rumen liquor supplemented by either CT or HT [65], it is evident that PC had a mechanism far from that suggested for tannin extracts. As such, tannins have been reported favouring *Prevotella* and its metabolism, which produces propionate and consumes hydrogen [65], on the contrary, propionate proportion, as well as *Prevotella* abundance, decreased in PC rumen liquor.

Chemical structure is decisive for activity in ruminal fermentation, and the antimicrobial activities of several EOCs with a wide chemical spectrum hence makes predicting the regulatory effect on rumen fermentation often difficult [13, 49]. At present, specific mechanisms of action have been proposed only for a few individual EOC compounds [3] and commercial products, which have already been validated for their mitigating effect in vivo (such as the product used for PC treatment) but were limitedly characterised for the mechanisms of action [10, 11]. In the present study, the severe defaunation caused by PC might partly explain the significant reduction in the production of ammonia, methane and total VFA (−17%) in comparison to NC. Moreover, the decline in methane formation with PC was associated with the lowest hydrogen concentration in the gas phase of all groups corroborating the explanation for the methane mitigation achieved with this product [61]. The ciliate protozoa may represent up to 50% of the microbial biomass in the rumen [66], in which they engulf organic matter particles and bacteria, and actively degrade fibre and other nutrients to produce VFA (particularly acetate and butyrate) and large amounts of hydrogen [1, 66, 67]. The latter is the reason for intensive associations of methanogens with the protozoa. Indeed, in the present study, archaeal abundance was lower in PC in comparison to Q-2 and C-10. The highly disruptive effect on the protozoa population as found with PC was not previously reported with Agolin Ruminant in vitro, at least not at this severity (only −15% according to a meta-analysis [10]). Still, a recent study proposed the change of relative abundance of protozoa as the principal mechanism of Agolin Ruminant in reducing in vivo methane [11]; besides, they did not report a significant effect on Archaea, which might have furtherly supported their methane reduction (−8.8% CH_4_/kg DM intake, [11]) and despite the present findings.

Compared to PC, dDM and dOM (expressed as % of OM supplied) were greater for Q-2 and C-10, whereas the degradability of NDF was smaller (Q-2) or tended to be smaller (C-10). Overall, this pattern of degradability was probably due to the inhibitory effect of PC on microorganisms degrading non-structural carbohydrates, including protozoa. As a matter of fact, *Butyrivibrio, Lachnospira* and *Treponema* (from 2 to 5-fold higher in PC than in Q-2 and C-10), which are known as fibre degrading bacteria [1, 49], were reported herein as having a higher relative abundance. However, it must be noted that NDF degradability was reduced by all treatments compared to NC, and, in the case of PC, this might have been partially due to the lower abundances of the *Fibrobacter* genus (−95%) and the Prevotellaceae family (−28%) in comparison to Q-2 and C-10. On the other hand, fungi relative abundance seemed not directly involved. Overall, the depression of feed degradation caused by PC and Q-2 compared to C-10 and NC was supported by the concomitant depression of total VFA produced. Unlike the present study, previous in vivo and in vitro studies [9, 10, 68] did not find a significant effect of Agolin on VFA production, maybe due to the lower concentration tested. The lowest molar proportion of propionate was found for PC, confirming what was previously reported by Pirondini et al. [68], using Agolin Ruminant. The lowest proportion of acetate was associated with the Q-2 treatment. Consequently, the acetate-to-propionate ratio was highest with PC, pointing towards a lower energetic value of the substrates obtained from fermentation in the animal. This modification of the VFA profile by PC was associated with a decrease in the succinate producers (i.e., *Fibrobacter*), usually considered to be involved in the fermentative pathway of propionate [69]. It is known that the effects on molar proportions of VFA vary between types of EO supplemented [15, 16], but the present study indicates that duration of the fermentation, diet composition and especially EOC dosage affects the exhibition and extent of such effects in vitro.

Some of the effects on nutrient degradability were not supported by the expected changes in abundance of the respective families or genera of bacteria or fungi. However, it has to be noted that the applied sequencing cannot define enough accurately the abundance of targeted species of individual microbe species. Therefore, a decrease in key fibrolytic bacteria species reported previously [60, 70] could not sufficiently be distinguished. In addition, the complex interaction of the EOC with the tannins, and of CT and HT with the feed particles, as they could have been complexed nutrients differently, might have caused differences in fibre degradability, methane and ammonia formation, which are not clearly reflected in the composition of the microbial communities [48].

Finally, the biodiversity (both richness and evenness) of the prokaryotes was highly reduced by PC (Fig. 1), if compared to both Q-2 and C-10 treatments, probably due to the far-going defaunation [71]. A similar, however less severe, trend was also reported for fungal richness (Fig. 2). Similarly, a recent study reported a lower α-diversity of rumen microbiome of dairy cows supplemented with 1 g/d of Agolin Ruminant [11]. The robustness and resilience of microbiota is a biomarker for animal health, and it increases with higher α-diversity and network complexity [72]. Thus, if the present in vitro data will be confirmed by in vivo experiments, the severe defaunation observed in the present study at the dosage of Agolin Ruminant used could cause substantial changes in the microbiota community and an undesired decline of the bacterial and fungal richness.

## Conclusions

The present study demonstrated that the selected mixtures of either quebracho (Q-2) or chestnut tannins (C-10) and blends of three essential oils each have the potential to mitigate absolute methane and ammonia formation in vitro over a period of 10 d. The Q-2 was effective in mitigating ammonia formation, whereas the C-10 mitigated more efficiently methane formation. The levels of either ammonia or methane mitigation of the respectively more efficient one of two mixtures were similar to that of the positive control, containing a blend of three essential oils but no tannins. Different from the PC, C-10 seemed to affect the microbiota metabolism and composition to a lesser extent as it did not affect total VFA production and the composition of bacterial and fungal communities, whereas Q-2 had effects more pronounced but still not as severe as those of PC. It seems that the action of the PC was mostly mediated by a severe defaunation and the consequent reduction of the biodiversity of prokaryotes and a slight reduction of fungal richness. Although with all supplements part of the mitigating effects was caused by reductions in dOM, dNDF or total VFA, with some differences, further in vivo studies are needed to assess whether this is the sole reason for the mitigation potential of Q-2 and C-10 or other still unknown mechanisms of mitigation might prevail over adverse effects, as it was reported for PC in previous in vivo studies. Furthermore, the mode of action of the tannin-essential oil mixtures on rumen lipid metabolism has to be clarified further, as ruminal biohydrogenation is important for ruminant-source food quality. This can for instance accomplished by considering the relationships of the microbial community with targeted ruminal biomarkers.

### Supplementary Information

**Additional file 1: Fig. S1.** Rarefaction curves based on the number of bacterial sequences (sample size) and the number of zOTUs in each sample.** Fig. S2.** Rarefaction curves based on the number of fungal sequences (sample size) and the number of zOTUs in each sample.** Fig. S3.** Non-metric multidimensional scaling (NMDS) plot based on not weighted UniFrac distances of bacterial and fungal communities.** Table S1.** Effect of the tannin-essential oil mixtures Q-2 and C-10 compared to negative control (NC) and positive control (PC) on the relative abundance of bacterial communities at family level.** Table S2.** Effect of the tannin-essential oil mixtures Q-2 and C-10 compared to negative control (NC) and positive control (PC) on the relative abundance of bacterial communities at genus level.** Table S3.** Effect of the tannin-essential oil mixtures Q-2 and C-10 compared to negative control (NC) and positive control (PC) on the relative abundance of fungal communities at family level.** Table S4.** Effect of the tannin-essential oil mixtures Q-2 and C-10 compared to negative control (NC) and positive control (PC) on the relative abundance of fungal communities at genus level.

## Data Availability

All data from this study are available from the corresponding author upon reasonable request. Sequencing data are available at the National Center for Biotechnology Information (NCBI), BioProject number PRJNA1032414.

## Abbreviations

ADF: Acid detergent fibre
ADL: Acid detergent lignin
C-10: Experimental treatment containing chestnut extract and EOC blend 10
CH_4_: Methane
CO_2_: Carbon dioxide
CP: Crude protein
CT: Condensed tannins
dCP: Degradability of CP calculated from the difference of supply and residue
dDM: Degradability of DM calculated from the difference of supply and residue
DM: Dry Matter
dNDF: Degradability of NDF calculated from the difference of supply and residue
dNDF_DM_: Degradability of NDF calculated from the difference of supply and residue and related to DM supplied
dOM: Degradability of OM calculated from the difference of supply and residue
dOM_DM_: Degradability of OM calculated from the difference of supply and residue and related to DM supplied
EO: Essential oils
EOC: Essential oil compounds
GP: Gas production
H_2_: Hydrogen
HT: Hydrolysable tannins
LSU: 26S Ribosomal large sub unit
NC: Negative control
NDF: Neutral detergent fibre
NMDS: Non-metric multidimensional scaling
OM: Organic matter
OM: Organic matter
PC: Positive control
PERMANOVA: Permutational multivariate analysis of variance
Q-2: Experimental treatment containing quebracho extract and EOC blend 2
Rusitec: Rusitec simulation technique
VFA: Volatile fatty acids
zOTU: Zero-radius operational taxonomic units
