# Supplementary material for: Evaluation of ruminal methane and ammonia formation and microbiota composition as affected by supplements based on mixtures of tannins and essential oils using Rusitec
Source: J Anim Sci Biotechnol. 2024 Apr 2;15:48. doi: 10.1186/s40104-024-01005-8 (PMC10986001; doi:10.1186/s40104-024-01005-8)
Supplement: Supplementary file 1 — Additional file 1: Fig. S1. Rarefaction curves based on the number of bacterial sequences (sample size) and the number of zOTUs in each sample. Fig. S2. Rarefaction curves based on the number of fungal sequences (sample size) and the number of zOTUs in each sample. Fig. S3. Non-metric multidimensional scaling (NMDS) plot based on not weighted UniFrac distances of bacterial and fungal communities. Table S1. Effect of the tannin-essential oil mixtures Q-2 and C-10 compared to negative control (NC) and positive control (PC) on the relative abundance of bacterial communities at family level. Table S2. Effect of the tannin-essential oil mixtures Q-2 and C-10 compared to negative control (NC) and positive control (PC) on the relative abundance of bacterial communities at genus level. Table S3. Effect of the tannin-essential oil mixtures Q-2 and C-10 compared to negative control (NC) and positive control (PC) on the relative abundance of fungal communities at family level. Table S4. Effect of the tannin-essential oil mixtures Q-2 and C-10 compared to negative control (NC) and positive control (PC) on the relative abundance of fungal communities at genus level. [file 40104_2024_1005_MOESM1_ESM.docx]

**Supplementary material to**

**Evaluation of ruminal methane and ammonia formation and microbiota composition as affected by supplements based on mixtures of tannins and essential oils using Rusitec**

Giulia Foggi*, Melissa Terranova, Matteo Daghio, Sergej L. Amelchanka, Giuseppe Conte, Simon Ineichen, Monica Agnolucci, Carlo Viti, Alberto Mantino, Arianna Buccioni, Michael Kreuzer and Marcello Mele

Correspondance: [giulia.foggi@agr.unipi.it](mailto:giulia.foggi@agr.unipi.it)

Full list of author information is available at the end of the main article


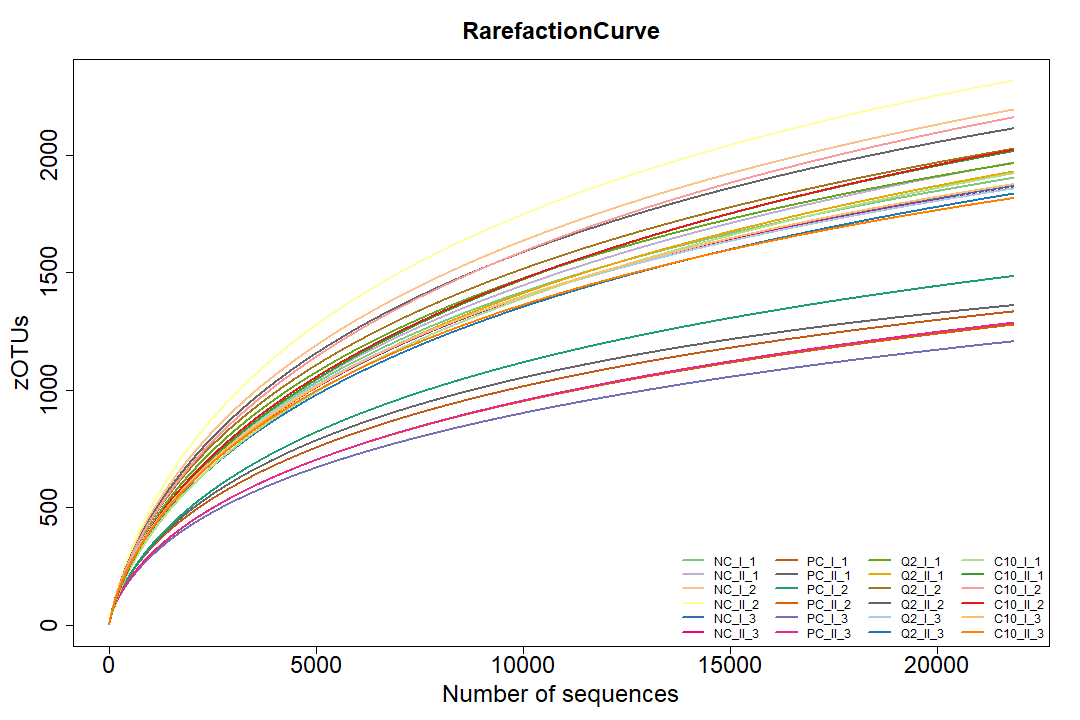


**Fig. S1** Rarefaction curves based on the number of bacterial sequences (sample size) and the number of zOTUs in each sample. NC, negative control; PC, positive control; Q-2, supplement Q-2; C-10, supplement C-10. Roman number indicate different samples within a run and Arabic number indicate the run.


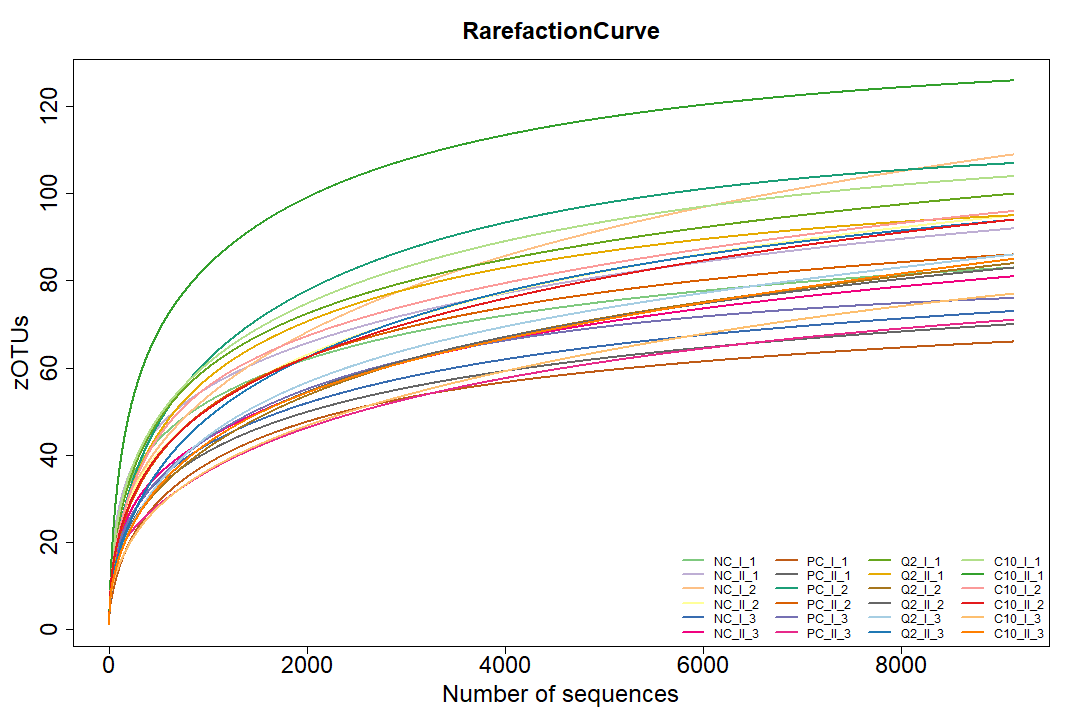


**Fig. S2** Rarefaction curves based on the number of fungal sequences (sample size) and the number of zOTUs in each sample. NC, negative control; PC, positive control; Q-2, supplement Q-2; C-10, supplement C-10. Roman number indicate different samples within a run and Arabic number indicate the run.

1. Bacteria (B) Fungi


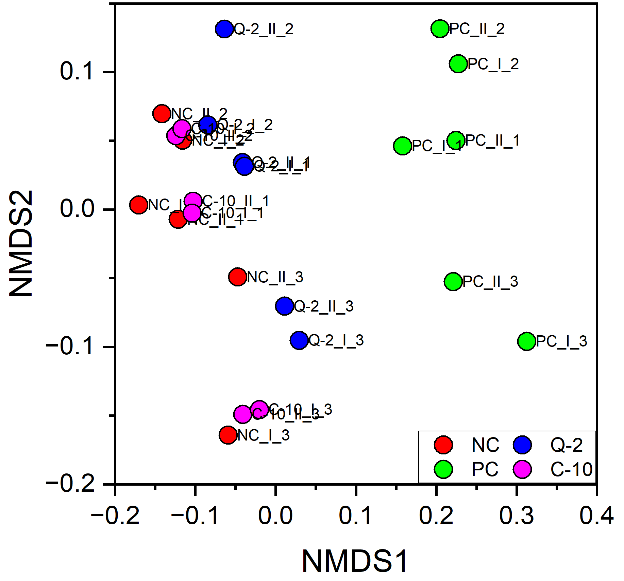

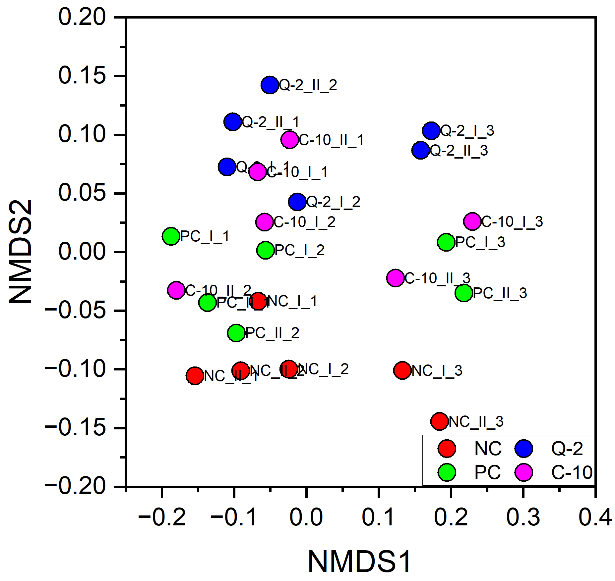


**Fig. S3** Non-metric multidimensional scaling (NMDS) plot based on not weighted Unifrac distances of bacterial (A) and fungal (B) communities. NC, negative control; PC, positive control, Q2, supplement Q-2; C10, supplement C-10. Roman number indicate different samples within a run and Arabic number indicate the run.

**Table S1**. Effect of the tannin-essential oil mixtures Q-2 and C-10 compared to negative control (NC) and positive control (PC) on the relative abundance of bacterial communities at family level.

|  | NC | | | PC | | | Q-2 | | | C-10 | | |  |
| --- | --- | --- | --- | --- | --- | --- | --- | --- | --- | --- | --- | --- | --- |
| Family | Average (%) | St. Dev. |  | Average (%) | St. Dev. |  | Average (%) | St. Dev. |  | Average (%) | St. Dev. |  | *P* |
| *Acidaminococcaceae* | 1.279 | 0.869 |  | 2.155 | 0.980 |  | 1.195 | 0.424 |  | 1.145 | 0.488 |  | 0.156 |
| *Anaeroplasmataceae* | 1.096 | 0.390 |  | 2.109 | 0.839 |  | 0.847 | 0.179 |  | 1.104 | 0.664 |  | 0.059 |
| *Bacteroidaceae* | 0.400 | 0.309 |  | 0.193 | 0.165 |  | 0.700 | 0.344 |  | 0.436 | 0.201 |  | 0.113 |
| *Bifidobacteriaceae* | 0.217 ^b^ | 0.184 |  | 0.850 ^a^ | 0.158 |  | 0.249 ^b^ | 0.113 |  | 0.217 ^b^ | 0.120 |  | 0.004 |
| *Clostridiales_Incertae_Sedis_XIII* | 0.990 | 1.028 |  | 0.153 | 0.124 |  | 0.751 | 0.827 |  | 0.854 | 0.976 |  | 0.061 |
| *Endomicrobiaceae* | 0.566 ^a^ | 0.227 |  | 0.001 ^c^ | 0.002 |  | 0.169 ^bc^ | 0.066 |  | 0.454 ^ab^ | 0.189 |  | 0.000 |
| *Erysipelotrichaceae* | 0.256 ^b^ | 0.139 |  | 1.211 ^a^ | 0.901 |  | 0.361 ^ab^ | 0.230 |  | 0.371 ^ab^ | 0.194 |  | 0.027 |
| *Fibrobacteraceae* | 4.165 ^a^ | 1.082 |  | 0.137 ^b^ | 0.121 |  | 1.481 ^ab^ | 1.372 |  | 3.883 ^ab^ | 2.576 |  | 0.001 |
| *Lachnospiraceae* | 5.113 ^c^ | 1.025 |  | 13.602 ^a^ | 1.606 |  | 8.819 ^ab^ | 1.628 |  | 6.166 ^bc^ | 1.108 |  | 0.000 |
| *Lactobacillaceae* | 5.206 | 2.181 |  | 9.390 | 2.223 |  | 8.681 | 1.697 |  | 7.766 | 3.085 |  | 0.058 |
| *Muribaculaceae* | 0.364 ^c^ | 0.206 |  | 0.002 ^b^ | 0.003 |  | 0.332 ^ab^ | 0.363 |  | 0.296 ^ab^ | 0.278 |  | 0.004 |
| *Prevotellaceae* | 23.128 ^a^ | 2.188 |  | 15.518 ^b^ | 3.466 |  | 20.374 ^ab^ | 1.698 |  | 23.170 ^a^ | 1.271 |  | 0.002 |
| *Ruminococcaceae* | 4.066 | 1.349 |  | 3.386 | 0.867 |  | 3.653 | 0.695 |  | 3.299 | 0.793 |  | 0.707 |
| *Selenomonadaceae* | 2.903 | 0.856 |  | 1.827 | 0.355 |  | 1.790 | 0.826 |  | 2.505 | 1.027 |  | 0.106 |
| *Sphaerochaetaceae* | 0.821 ^a^ | 0.489 |  | 0.065 ^b^ | 0.044 |  | 0.965 ^a^ | 0.429 |  | 0.904 ^a^ | 0.437 |  | 0.004 |
| *Spirochaetaceae* | 3.488 ^b^ | 0.678 |  | 6.651 ^a^ | 1.788 |  | 3.922 ^b^ | 0.540 |  | 3.735 ^b^ | 1.494 |  | 0.004 |
| *Streptococcaceae* | 0.675 ^b^ | 0.409 |  | 2.539 ^a^ | 0.482 |  | 2.282 ^a^ | 1.359 |  | 1.487 ^ab^ | 0.607 |  | 0.003 |
| *Succinivibrionaceae* | 2.321 ^ab^ | 2.379 |  | 0.282 ^b^ | 0.140 |  | 0.758 ^ab^ | 0.980 |  | 2.461 ^a^ | 2.819 |  | 0.017 |
| *Sutterellaceae* | 0.275 | 0.213 |  | 0.168 | 0.100 |  | 0.152 | 0.106 |  | 0.140 | 0.092 |  | 0.416 |
| *Veillonellaceae* | 2.827 | 3.214 |  | 4.432 | 3.488 |  | 2.406 | 2.559 |  | 2.642 | 2.404 |  | 0.655 |
| Other families | 1.920 | 0.700 |  | 1.577 | 0.340 |  | 1.640 | 0.310 |  | 1.703 | 0.419 |  |  |
| Unknown | 37.924 | 7.919 |  | 33.751 | 4.041 |  | 38.473 | 4.571 |  | 35.262 | 4.889 |  |  |

**Table S2.** Effect of the tannin-essential oil mixtures Q-2 and C-10 compared to negative control (NC) and positive control (PC) on the relative abundance of bacterial communities at genera level.

|  | NC | | | PC | | | Q-2 | | | C-10 | | |  |
| --- | --- | --- | --- | --- | --- | --- | --- | --- | --- | --- | --- | --- | --- |
| Genus | Average (%) |  | St. Dev. | Average (%) |  | St. Dev. | Average (%) |  | St. Dev. | Average (%) |  | St. Dev. | *P* |
| *Acidaminococcus* | 0.317 |  | 0.436 | 0.557 |  | 0.729 | 0.172 |  | 0.235 | 0.269 |  | 0.368 | 0.795 |
| *Anaeroplasma* | 0.800 |  | 0.315 | 1.567 |  | 0.865 | 0.563 |  | 0.133 | 0.814 |  | 0.476 | 0.069 |
| *Anaerovibrio* | 0.723 | ^a^ | 0.270 | 0.302 | ^b^ | 0.162 | 0.745 | ^a^ | 0.138 | 0.737 | ^a^ | 0.153 | 0.005 |
| *Asteroleplasma* | 0.295 |  | 0.106 | 0.542 |  | 0.129 | 0.285 |  | 0.173 | 0.290 |  | 0.193 | 0.047 |
| *Bacilliculturomica* | 0.771 |  | 1.046 | 0.021 |  | 0.024 | 0.521 |  | 0.776 | 0.626 |  | 0.881 | 0.165 |
| *Butyrivibrio* | 0.620 | ^b^ | 0.181 | 2.962 | ^a^ | 1.664 | 1.506 | ^ab^ | 1.092 | 0.778 | ^b^ | 0.413 | 0.005 |
| *Clostridium_XlVa* | 0.408 |  | 0.289 | 0.225 |  | 0.105 | 0.403 |  | 0.172 | 0.404 |  | 0.279 | 0.350 |
| *Duodenibacillus* | 0.275 |  | 0.213 | 0.168 |  | 0.100 | 0.152 |  | 0.106 | 0.140 |  | 0.092 | 0.416 |
| *Endomicrobium* | 0.566 | ^a^ | 0.227 | 0.001 | ^c^ | 0.002 | 0.169 | ^bc^ | 0.066 | 0.454 | ^ab^ | 0.189 | 0.000 |
| *Fibrobacter* | 4.165 | ^a^ | 1.082 | 0.137 | ^b^ | 0.121 | 1.481 | ^ab^ | 1.372 | 3.883 | ^a^ | 2.576 | 0.001 |
| *Intestinibaculum* | 0.000 |  | 0.000 | 0.157 |  | 0.362 | 0.020 |  | 0.044 | 0.021 |  | 0.033 | 0.801 |
| *Lachnospira* | 0.132 | ^b^ | 0.116 | 1.539 | ^a^ | 0.407 | 0.459 | ^ab^ | 0.281 | 0.344 | ^b^ | 0.355 | 0.001 |
| *Lactobacillus* | 2.406 |  | 1.722 | 4.881 |  | 1.246 | 4.262 |  | 2.390 | 3.182 |  | 2.151 | 0.150 |
| *Ligilactobacillus* | 0.172 |  | 0.106 | 0.140 |  | 0.139 | 0.174 |  | 0.168 | 0.321 |  | 0.324 | 0.706 |
| *Limosilactobacillus* | 2.628 |  | 1.151 | 4.368 |  | 1.124 | 4.246 |  | 3.063 | 4.263 |  | 3.390 | 0.288 |
| *Mediterranea* | 0.062 | ^ab^ | 0.045 | 0.089 | ^b^ | 0.089 | 0.341 | ^a^ | 0.352 | 0.108 | ^ab^ | 0.064 | 0.039 |
| *Megasphaera* | 2.760 |  | 3.265 | 4.422 |  | 3.480 | 2.380 |  | 2.565 | 2.627 |  | 2.404 | 0.635 |
| *Paramuribaculum* | 0.182 | ^a^ | 0.282 | 0.001 | ^b^ | 0.002 | 0.279 | ^a^ | 0.381 | 0.182 | ^a^ | 0.310 | 0.011 |
| *Paraprevotella* | 0.536 |  | 0.590 | 0.098 |  | 0.083 | 0.103 |  | 0.072 | 0.450 |  | 0.459 | 0.206 |
| *Prevotella* | 21.842 | ^a^ | 2.105 | 14.890 | ^b^ | 3.522 | 19.288 | ^ab^ | 1.823 | 21.745 | ^a^ | 1.777 | 0.002 |
| *Pseudobutyrivibrio* | 0.208 | ^c^ | 0.053 | 0.922 | ^a^ | 0.206 | 0.555 | ^ab^ | 0.096 | 0.329 | ^bc^ | 0.074 | 0.000 |
| *Pseudoscardovia* | 0.206 | ^b^ | 0.187 | 0.844 | ^a^ | 0.158 | 0.246 | ^b^ | 0.114 | 0.215 | ^b^ | 0.118 | 0.004 |
| *Roseburia* | 0.002 |  | 0.002 | 0.152 |  | 0.258 | 0.329 |  | 0.626 | 0.056 |  | 0.075 | 0.526 |
| *Ruminococcus* | 0.157 | ^c^ | 0.045 | 0.666 | ^a^ | 0.187 | 0.363 | ^ab^ | 0.094 | 0.187 | ^bc^ | 0.034 | 0.000 |
| *Schwartzia* | 0.559 | ^ab^ | 0.184 | 0.905 | ^a^ | 0.163 | 0.415 | ^b^ | 0.268 | 0.442 | ^b^ | 0.322 | 0.021 |
| *Selenomonas* | 0.637 |  | 0.663 | 0.299 |  | 0.394 | 0.143 |  | 0.149 | 0.359 |  | 0.171 | 0.109 |
| *Streptococcus* | 0.675 | ^b^ | 0.409 | 2.539 | ^a^ | 0.482 | 2.282 | ^a^ | 1.359 | 1.487 | ^ab^ | 0.607 | 0.003 |
| *Succiniclasticum* | 0.962 |  | 0.468 | 1.598 |  | 0.699 | 1.023 |  | 0.236 | 0.876 |  | 0.264 | 0.245 |
| *Succinivibrio* | 2.112 |  | 2.372 | 0.123 |  | 0.106 | 0.627 |  | 1.009 | 2.183 |  | 2.826 | 0.032 |
| *Treponema* | 3.479 | ^b^ | 0.668 | 6.648 | ^a^ | 1.791 | 3.902 | ^b^ | 0.536 | 3.724 | ^b^ | 1.490 | 0.004 |
| Other genera | 2.634 |  | 0.741 | 2.376 |  | 0.428 | 2.283 |  | 0.491 | 2.371 |  | 0.573 |  |
| Unknown | 48.706 |  | 8.935 | 45.859 |  | 5.426 | 50.285 |  | 4.885 | 46.135 |  | 5.444 |  |

**Table S3.** Effect of the tannin-essential oil mixtures Q-2 and C-10 compared to negative control (NC) and positive control (PC) on the relative abundance of fungal communities at family level.

|  | NC | | | PC | | | Q-2 | | | C-10 | | |  |
| --- | --- | --- | --- | --- | --- | --- | --- | --- | --- | --- | --- | --- | --- |
| Family | Average (%) |  | St. Dev. | Average (%) |  | St. Dev. | Average (%) |  | St. Dev. |  |  | St. Dev. | *P* |
| *Cystobasidiaceae* | 0.046 |  | 0.035 | 0.126 |  | 0.143 | 0.096 |  | 0.106 | 0.167 |  | 0.241 | 0.420 |
| *Davidiellaceae* | 0.040 | ^b^ | 0.029 | 0.087 | ^ab^ | 0.069 | 0.195 | ^a^ | 0.125 | 0.203 | ^ab^ | 0.213 | 0.027 |
| *Didymellaceae* | 0.043 | ^b^ | 0.038 | 0.111 | ^ab^ | 0.067 | 0.300 | ^a^ | 0.146 | 0.368 | ^a^ | 0.439 | 0.003 |
| *Dothioraceae* | 0.002 |  | 0.004 | N.D. |  | N.D. | 0.134 |  | 0.316 | 0.017 |  | 0.034 | 0.082 |
| *Neocallimastigaceae* | 86.063 |  | 11.143 | 85.190 |  | 11.372 | 83.689 |  | 7.873 | 84.630 |  | 7.887 | 0.981 |
| *Pleosporaceae* | 0.019 |  | 0.025 | 0.026 |  | 0.021 | 0.092 |  | 0.064 | 0.172 |  | 0.292 | 0.096 |
| *Saccharomycetaceae* | 0.027 |  | 0.042 | 0.026 |  | 0.040 | 0.009 |  | 0.015 | 0.152 |  | 0.272 | 0.657 |
| *Sporidiobolales_incertae_sedis* | 0.218 |  | 0.083 | 0.241 |  | 0.173 | 0.566 |  | 0.410 | 0.755 |  | 1.211 | 0.119 |
| *Tremellaceae* | 0.244 | ^b^ | 0.073 | 0.510 | ^ab^ | 0.393 | 0.900 | ^a^ | 0.542 | 1.212 | ^ab^ | 1.571 | 0.045 |
| *Trichocomaceae* | 0.524 |  | 0.501 | 0.725 |  | 0.483 | 0.474 |  | 0.359 | 0.581 |  | 0.529 | 0.809 |
| *Tricholomataceae* | 0.007 |  | 0.009 | 0.004 |  | 0.006 | 0.165 |  | 0.397 | 0.003 |  | 0.005 | 0.870 |
| *Wallemiaceae* | 11.931 |  | 10.931 | 11.565 |  | 9.829 | 10.127 |  | 8.395 | 8.896 |  | 5.470 | 0.982 |
| Other families | 0.392 |  | 0.186 | 0.496 |  | 0.269 | 0.683 |  | 0.368 | 0.527 |  | 0.577 |  |
| Unknown | 0.444 |  | 0.133 | 0.893 |  | 0.639 | 2.571 |  | 1.442 | 2.314 |  | 3.203 |  |

**Table S4.** Effect of the tannin-essential oil mixtures Q-2 and C-10 compared to negative control (NC) and positive control (PC) on the relative abundance of fungal communities at genera level.

|  | NC | | | PC | | | Q-2 | | | C-10 | | |  |
| --- | --- | --- | --- | --- | --- | --- | --- | --- | --- | --- | --- | --- | --- |
| Genus | Average (%) |  | St. Dev. | Average (%) |  | St. Dev. | Average (%) |  | St. Dev. |  |  | St. Dev. | *P* |
| *Anaeromyces* | 0.214 |  | 0.281 | 0.002 |  | 0.006 | 0.002 |  | 0.006 | 0.049 |  | 0.069 | 0.054 |
| *Caecomyces* | 6.417 |  | 4.053 | 3.116 |  | 2.834 | 4.862 |  | 4.732 | 6.381 |  | 5.083 | 0.283 |
| *Clitocybe* | 0.001 |  | 0.002 | N.D. |  | N.D. | 0.163 |  | 0.398 | N.D. |  | N.D. | 0.262 |
| *Cryptococcus* | 0.206 | ^b^ | 0.060 | 0.482 | ^ab^ | 0.381 | 0.704 | ^a^ | 0.446 | 0.904 | ^ab^ | 1.154 | 0.045 |
| *Davidiella* | 0.032 | ^b^ | 0.022 | 0.071 | ^ab^ | 0.069 | 0.189 | ^a^ | 0.128 | 0.200 | ^a^ | 0.214 | 0.011 |
| *Eurotium* | 0.148 |  | 0.076 | 0.257 |  | 0.170 | 0.182 |  | 0.130 | 0.304 |  | 0.422 | 0.701 |
| *Neocallimastix* | 38.906 | ^b^ | 14.142 | 57.567 | ^ab^ | 20.500 | 68.639 | ^a^ | 8.318 | 58.148 | ^ab^ | 14.600 | 0.036 |
| *Orpinomyces* | 28.401 |  | 19.000 | 13.811 |  | 14.586 | 2.807 |  | 2.179 | 12.291 |  | 9.951 | 0.056 |
| *Saccharomyces* | 0.023 |  | 0.044 | 0.021 |  | 0.040 | 0.006 |  | 0.015 | 0.140 |  | 0.245 | 0.388 |
| *Sporidiobolus* | 0.218 |  | 0.083 | 0.241 |  | 0.173 | 0.566 |  | 0.410 | 0.755 |  | 1.211 | 0.119 |
| *Wallemia* | 11.931 |  | 10.931 | 11.565 |  | 9.829 | 10.127 |  | 8.395 | 8.896 |  | 5.470 | 0.982 |
| Other genera | 0.511 |  | 0.164 | 0.569 |  | 0.294 | 0.778 |  | 0.407 | 0.758 |  | 0.934 |  |
| Unknown | 12.993 |  | 3.640 | 12.298 |  | 11.360 | 10.973 |  | 4.045 | 11.173 |  | 4.863 |  |
